# Demographic and Epidemiological Contributions to Recent Trends in Cancer Incidence in Hong Kong

**DOI:** 10.3390/cancers13225727

**Published:** 2021-11-16

**Authors:** Irene Oi Ling Wong, Yan Ting Lam, Kwok Fai Lam, Benjamin John Cowling, Gabriel Matthew Leung

**Affiliations:** 1 School of Public Health, The University of Hong Kong, Hong Kong SAR, China; lamyant@hku.hk (Y.T.L.); bcowling@hku.hk (B.J.C.); gmleung@hku.hk (G.M.L.); 2 Department of Statistics and Actuarial Science, The University of Hong Kong, Hong Kong SAR, China; hrntlkf@hku.hk

**Keywords:** cancer, incidence, Chinese

## Abstract

**Simple Summary:**

Hong Kong has an ageing Chinese population with high life expectancy and a rising number of new cancer cases (156.5% increase for women and a 96% increase for men during the period 1983–2017). While both population growth and population ageing could contribute to this trend, it is unknown whether change in disease risk contributes to or inhibits this trend. In this study, we quantify the demographic and epidemiological contributions to this trend by disentangling the effect of these factors, finding that this increasing trend is mostly due to population growth (66.1% for women, 25.4% for men) and population ageing (95% for women, 119.4% for men), with changes in disease risk inhibiting this increasing trend (−4.5% for women, −48.8% for men).

**Abstract:**

Background: Hong Kong has an ageing Chinese population with high life expectancy and a rising number of cancer cases. While population ageing could lead to higher incidence, we aim to quantify the demographic and epidemiological contributions to this trend by disentangling the effect of these factors. Methods: We analysed secular trends of cancer incidences of all cancer sites combined, including the five top cancers in men and women in Hong Kong in 1983–2017, by disentangling effects of demographics (ageing population and population growth) and cancer risk/rate change using the RiskDiff methodology. Results: Overall, age-standardised incidences of all cancers combined in women and in men declined over the study period (−5.3% for women, −30.2% for men), but total incident cancer case counts increased dramatically (156.5% for women, 96% for men). This increase was primarily due to ageing and increasing population (95% age, 66.1% growth for women, and 119.4% age, 25.4% growth for men), while disease risk for all cancers combined has a decreasing trend (−4.5% for women and −48.8% for men). For the site-specific risk changes among the most five common cancer types, there were increases in risks of prostate and colorectal cancers in men, and breast, endometrial, and thyroid cancers in women. Conclusion: Demographic changes and ageing in our Chinese population resulted in a marked increase in the number of cancer diagnoses in Hong Kong in past decades. The surge in incident case counts overall is expected to stress the healthcare system in terms of the increased demand of healthcare professionals. Cancer surveillance should be enhanced in view of the growing demand from older patients and the cancer types with fast-increasing incidence rates in our population.

## 1. Introduction

Cancer ranks as a leading cause of death in the world, claiming 10.0 million lives in 2020 [1], and increasing economic burden worldwide [2]. The global increase in cancer burden is propelled largely by demographic changes in the population at risk and its association with etiologic risk factors, several of which are linked with socioeconomic development [3,4]. However, less attention has been given to the recent trend of cancers in Chinese population driven by both demographic and epidemiological variations.

Hong Kong, one of the most westernized and urbanized cities in China, has the world’s highest life expectancy and is experiencing a more rapid transition towards an ageing society [5]. The proportion of elderly above 65 years old in Hong Kong is projected to increase from 17% in 2016 to 29% in 2031 and 36% in 2056 [6], and the rate of ageing appears to be faster than other high-income countries [7]. Over half of the cancer cases in Hong Kong are diagnosed after the age of 60, showing that cancer is predominately a disease of the elderly [8], despite anecdotal observations of more cases of some cancers being diagnosed in younger patients. We postulate that the rapidly ageing population could affect burdens of different cancer types, and it could probably occur in other high-income transitioned countries with a similarly increasing life expectancy.

In this study, we analysed the secular disease trends of cancer incidences of all cancer sites combined and the five top cancers (ranked by the figures in 2017) for males and females in Hong Kong. We quantified the effects of demographic and epidemiological changes on the trends from 1983 to 2017 in Hong Kong by disentangling the effects of population structure (ageing population and population growth) and the change in cancer risk to major cancers using the RiskDiff methodology [9]. This decomposition of the changing disease burden into demographic and epidemiological components allows us to put the effectiveness of the relevant prevention strategies into perspective, and this information could be helpful for healthcare resource prioritization and better future projection of disease burden.

## 2. Materials and Methods

### 2.1. Data Sources

We retrieved age-specific cancer incidences for the top 5 male and female cancers (i.e., the International Classification of Diseases (ICD) codes ICD-10 C33-34, C18-21, C61, C22, C16, C50, C54, and C73) from 1983–2017, compiled from the Hong Kong Cancer Registry (HKCR) [10], where the HKCR is the most carefully validated data source for cancers in Hong Kong and is maintained according to the world standard of the International Agency for Research on Cancer (IARC), WHO. The top five male cancers were carcinoma in lung, colorectum, prostate, liver, and stomach, while the top five female cancers are breast, colorectum, lung, endometrium, and thyroid in 2017. These most common cancers were similar but in different orders when compared to the ranks worldwide. Around the world, the five most common cancers for men were lung, prostate, colorectal, stomach, and liver cancers, while breast, colorectal, lung, cervix uteri, and thyroid cancers occupied the top five in women [1].

We also obtained population figures, including age and sex distribution (from 1983 to 2017) from the Census and Statistics Department [6]. For a comparison with other high-income countries, we retrieved age-specific cancer incidences for the same set of cancers in Australia, Japan, Singapore, United Kingdom, and United States, as well as the corresponding population data from 1990 to 2017 from the Global Health Data Exchange, which is one of the world’s most comprehensive health-related open data source and is produced by the Institute for Health Metrics and Evaluation [11].

### 2.2. Statistical Analyses

To examine time trends in incidence rates, we estimated the age-standardised rates using the World Standard Population (WHO) in 2000. Segmented regression or joinpoint regression [12] was applied to break down the overall trend into several constituent linear trends and identify the breakpoints joining the linear constituents (i.e., inflection points), where disease rate vary significantly. The method characterizes the trends succinctly and permits us to statistically test for recent changes in trends. The annual percentage change (APCs) in each linear period was derived as the slope of the line in that period. The rate of change within every linear period was presented as APCs, and the overall changes between 1983–2017 were presented as average annual percentage change (AAPC), that is derived as the weighted average of the APCs over the time period studied.

We applied the RiskDiff methodology [9], initially proposed by Bashir and Estève [13], which partitions change in disease rates into epidemiological (i.e., population disease risks and diagnostic practices; alternatively, called as risk herein) and demographic (i.e., population size and population age structure) components [14]. The method first standardizes the two populations (i.e., in an earlier period and in a later period, or two consecutive years herein this study) to equal sizes (e.g., 100,000), and then applies the age-specific incidence rate in the earlier period to the population structure in the later period to assess the incidence changes due to population structure changes and cancer risk changes, leaving the effect of population size change exposed as the remainder. Additional technical details of the statistical methodology are listed in Supplementary Information S1.

For cross-country comparison, we selected high-income and transitioned countries including Australia, Japan, Singapore, UK, and US for the same cancer types in the period from 1990–2017 (where data were available), using the year of 1990 as the baseline. We applied the RiskDiff method and calculated the sex-specific attributable percentage changes in the number of cases of cancers from 1990 to 2017 due to differences in the risk, and the second component of those due to differences in demographic factor (i.e., population structure/age distribution and population size).

We also conducted a sensitivity test between the RiskDiff method and the similar method used in the Global Burden Disease Study [15], as Cheng et al. has a discussion of these two decomposition methodologies [16].

All statistical analyses were implemented using the R version 4.0.0 and Joinpoint Regression Program v4.6.0 from the National Cancer Institute (Bethesda, ML, USA).

## 3. Results

### 3.1. Trends of the Major Cancers in Hong Kong

**Figure 1:**
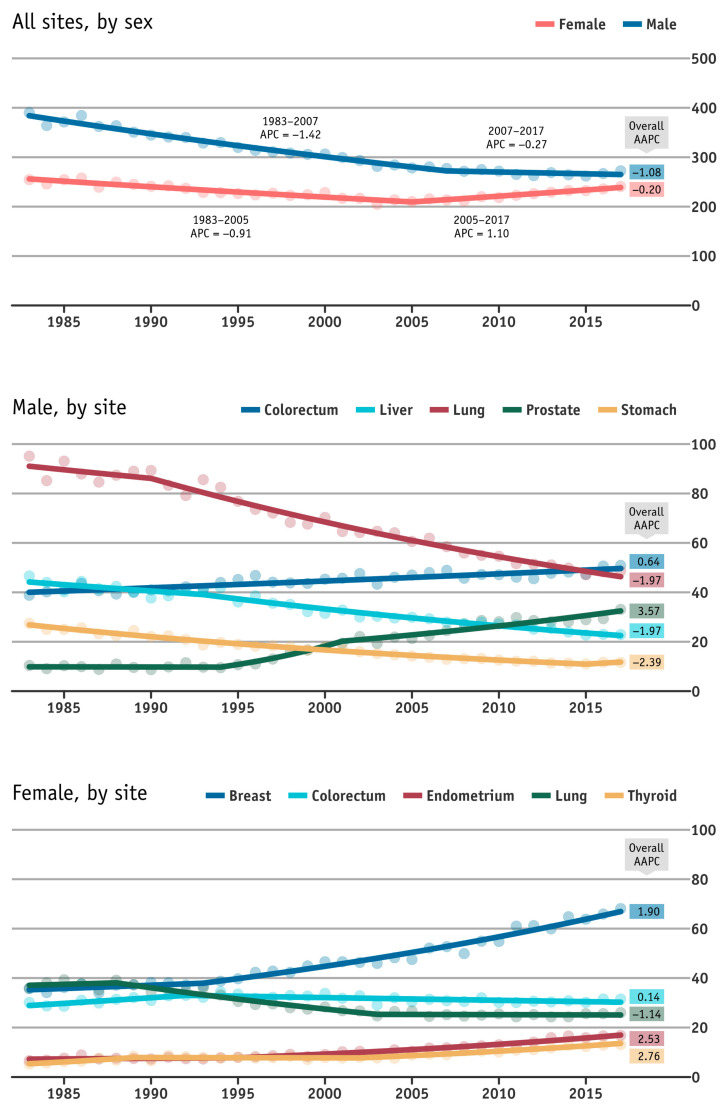
Annual age-standardised incidence rates for top five male/female cancers in Hong Kong from 1983 to 2017. The rates are age-adjusted according to the WHO standard population in 2000. Top five cancers are based on the figures from the Hong Kong Cancer Registry in 2017. (NB: average annual percentage change = AAPC; annual percentage change = APC).

Figure 1 shows the age-standardised incidence rate for all cancer sites combined, which are the male and female cancers in Hong Kong from 1983 to 2017. In 2017, the age-standardised rate of cancer overall was 258.0 per 100,000 people (272.5 for men and 240.8 for women) in Hong Kong, down from 313.1 per 100,000 people (390.1 for men and 254.5 for women) in 1983. The AAPC was observed to be −0.60% overall (−1.10% for men and −0.20% for women), which is an indication of decreasing overall disease risks with more noticeable decline among men in Hong Kong in past three decades. However, compared to the earlier years, there was an increasing trend for all sites combined for female cancers overall between 2005 and 2017 (APC = 1.1%), and a decreasing trend for all sites combined for male cancers overall between 2007 and 2017 (APC = −0.3%).

Disease rates varied by sex and sites over the years from 1983–2017 (Figure 1). Prostate cancer risks increased most rapidly and ranked at the top rate of increases among the more common cancer sites in men and women (AAPC = 3.57%), followed by other cancer sites including the female thyroid (AAPC = 2.76%), female endometrium (2.53%), female breast (1.90%), and colorectum (0.64% for male, 0.14% for female). On the other hand, male stomach cancer risk trended downward (AAPC = −2.39%), and male liver (−1.97%) and lung (−1.97% for men, −1.14% for women) also have lesser rates of decline.

### 3.2. Apportioned Number of New Cases into the Contribution from the Change in Population Risk, and Changes in Population Size and Age Structure Combined

**Figure 2:**
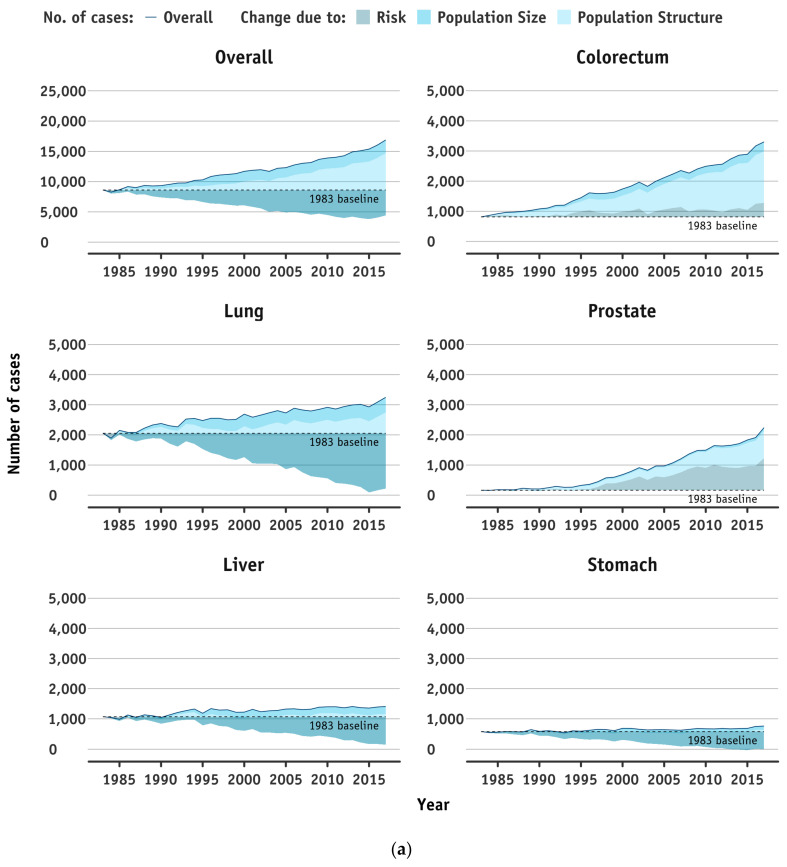

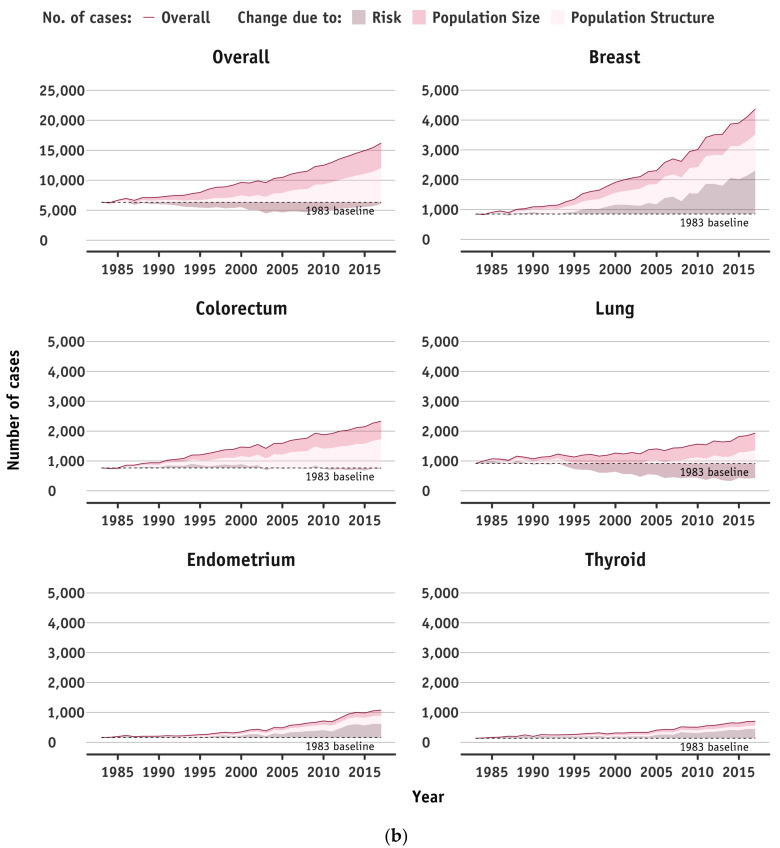
(**a**) Trends in number of incident cases for all cancer sites combined and top five cancers (ranked by 2017 figures) attributed to changes in disease risks and diagnostic practices (i.e., epidemiological components), and population size and structure (i.e., demographic components) for men in Hong Kong from 1983 to 2017. The solid line represents the overall number of incident cases, while the shaded areas represent the change in incident cases (compared to the 1983 baseline, dashed line) due to risk, population structure, and population size. (**b**) Trends in number of incident cases for all cancer sites combined and top five cancers (ranked by 2017 figures) attributed to changes in disease risks and diagnostic practices (i.e., epidemiological components), and population size and structure (i.e., demographic components) for women in Hong Kong from 1983 to 2017. The solid line represents the overall number of incident cases, while the shaded areas represent the change in incident cases (compared to the 1983 baseline, dashed line) due to risk, population structure, and population size.

**Figure 3:**
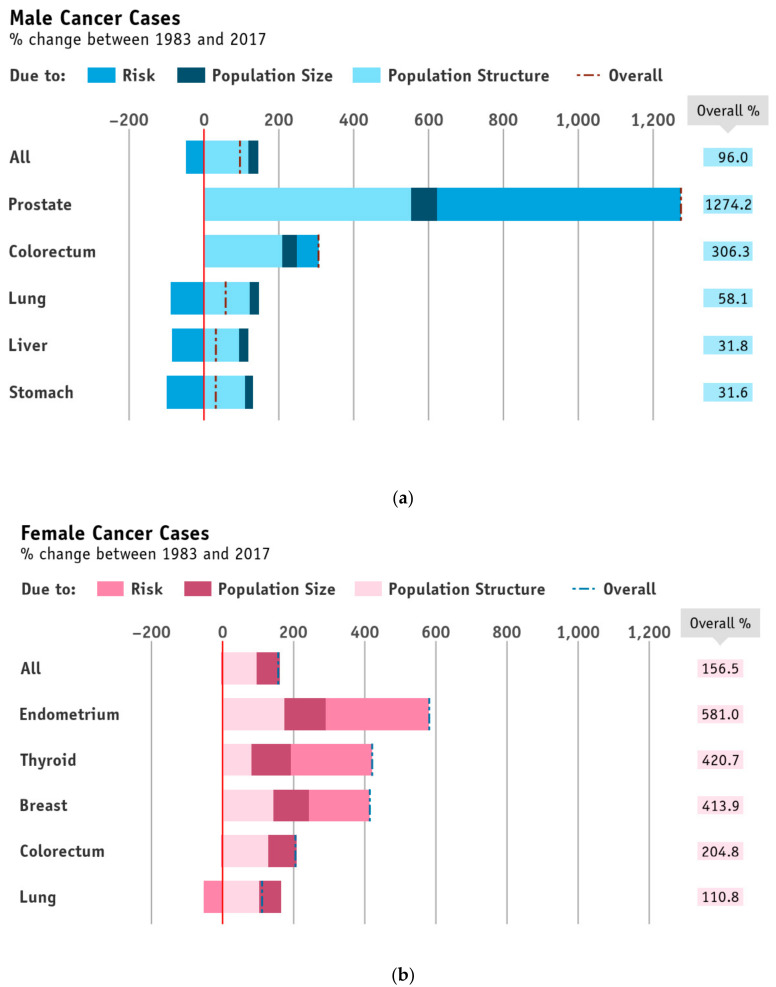
(**a**) Percentage changes in number of incident cases of the top five cancers (ranked by 2017 figures) in men in Hong Kong in 2017 attributed to population disease risk and diagnostic practices (i.e., epidemiological component), and population size and structure (i.e., demographic component), by sex, using 1983 as the baseline. Bars are ranked by increasing overall percentage changes. (**b**) Percentage changes in number of incident cases of the top five cancers (ranked by 2017 figures) in women in Hong Kong in 2017 attributed to population disease risk and diagnostic practices (i.e., epidemiological component), and population size and structure (i.e., demographic component), by sex, using 1983 as the baseline. Bars are ranked by increasing overall percentage changes.

Figure 2a,b and Figure 3a,b show the contribution to the changes in the number of incident cases by year of diagnosis, which are attributed to population disease risk and diagnostic practices (i.e., epidemiological component; alternatively, called as risk hereafter), and population size and structure (i.e., demographic component) by sex, between 1983 and 2017. Over the three decades, incident cases for all sites combined and the top individual cancers increased in men and women. The increases were primarily because of population growth and structure change, with positive or offset effects due to site-specific changes in risks. By 2017, new case counts (all sites) increased among women 156.5% (−4.5% risk, 95% age, and 66.1% growth) to 16,199 cases, and rose among men by 96% (−48.8% risk, 119.4% age, and 25.4% growth) to 16,876 cases. In parallel with the findings of AAPCs, decreases in disease risks were observed in all sites combined in both sexes. For the common cancers, overall trends in disease risks were declines in lung cancers in both sexes, as well as male liver and stomach cancers, while rising disease risks were found in several cancer sites, namely the prostate and colorectum for men, and the endometrium, thyroid, and breast for women.

We compared our attributable percentage change in the number of cases of cancers with the selected high-income and transitioned countries, including Australia, Japan, Singapore, UK, and US, for the same cancer types in the period 1990–2017 (Supplementary Figure S1). Barring male stomach cancer in Japan and the UK, and male lung cancer in the UK, the number of cases for the selected cancers in the countries have increased in the period; and changes in the number of cases attributed to size and population structure are all positive to a noticeable degree (except for change due to size in male prostate cancer in Japan).

In the sensitivity test on the decomposition method, we observed that the choice of decomposition methodology (i.e., the RiskDiff and GBD methods) had no material impact on our main findings (Supplementary Information S2).

## 4. Discussion

In our study, we evaluated the burden of cancers overall, and in the five major individual cancer sites for men and women in the Hong Kong Chinese population, by investigating their trends and assessing the contributions to the temporal disease changes due to demographic transition and cancer risks during 1983–2017. In the past three decades, age-standardised incidences of all cancers combined in men and women declined (AAPC = −1.1% in men, −0.2% in women), but overall incident case numbers increased dramatically in Hong Kong (cumulatively, a 96.0% and 156.5% increase in men and women, respectively). Upon decomposing the change into demographic and epidemiological components, we found that rises in incident cases (all sites) were mostly attributable to population growth and population structure (i.e., ageing population) in both sexes (119.4% age and 25.4% growth in men; 95.0% age and 66.1% growth in women) which was offset by a gradual decline in cancer risks/rates overall in males (−48.8% risk) and females (−4.5% risk) over time. For the site-specific risk changes, there was a drastic decline in risks in male lung, liver, and stomach cancers, as well as for female lung cancer; while there was significant rise in rates of male carcinoma in prostate and colorectum, and female carcinoma in endometrium, thyroid, and breast.

While the demographic component of cancer burden change is mostly associated with population ageing and population growth, the disease in the risk component would likely be associated with extended cancer screening, as in the case of breast cancer [17], prostate cancer [18], colorectal cancer, and improvements in diagnosis, as in the case of thyroid cancer [19]; declines in cancer incidence rates related to vaccination programmes, as in the case of hepatitis B vaccination and liver cancer [20]; reduction in smoking prevalence in the case of lung cancer [21]; and screening and eradication therapy of Helicobacter pylori infections as well as improvements in the food preservation and storage, in the case of stomach cancer [22,23,24]. Well-established associations would also explain change in disease risks. For instance, for breast cancer, unhealthy lifestyles and reproductive risk factors, such as women having fewer children at an older age, could contribute to the increase [25]. For endometrial cancer, increases in incidence may mainly occur as a consequence of rising obesity prevalence [26,27]. We therefore suggest primary prevention and early cancer detection as ways to combat the trends.

Epidemiological transitions, accounting for the receding of infectious diseases and emergence of degenerative conditions due to improvements in public health and sanitation, might also offer a plausible explanation to changes in disease patterns or population cancer risks [28]. According to the WHO report, as countries become better developed, cancer burdens tended to increase, and in particular, cancers related to industrialized lifestyles (e.g., colorectal, breast, and prostate) tended to displace infection-related cancers (e.g., cervical, liver, and stomach) [29]. Hong Kong has experienced rapid socio-economic transition in the previous century [30]. Our earlier study identified cohort-specific reductions in the incidence of infection-related cancers and rise in hormonally-modulated cancers (e.g., breast and prostate) in Hong Kong, and postulated the effect to be due to economic development [30]. This relevancy of epidemiological transition theory on change in population cancer risks might interpret our findings in the rise of incidence rates of colorectal and prostate cancer in men and female hormonally-modulated cancers, such as female breast, endometrial, and thyroid cancers, and the decrease in rates of liver and stomach cancers.

For cross-country comparison, new case counts of the cancer types increased in high income and transitioned countries, including Australia, Japan, Singapore, UK, and US from 1990–2017, and most of these disease trends can be accounted by ageing and growing populations. Of the major cancers studied, we see that male incident prostate cancers in Hong Kong have increased much more significantly (at an ~11-fold increase since 1990) than in other countries. Female breast and thyroid incident cancers in Hong Kong also have a larger increase (at a 4-fold increase and 3.6-fold increase, respectively) than other high-income countries studied. Further analytic studies would be warranted.

Other earlier studies have also speculated similar effects of population growth and population ageing on the disease burden of cancer in the United States [31], Europe [32], and China [33]. For instance, cancer incidence in the United States is projected to increase by 67% in the elderlies (age > 65) from 2010 to 2030; while only a 11% increase is expected for young adults [31]. In Nordic countries, the total number of cancer cases is expected to increase by 49% between 1993–1997 and 2018–2022, with 45% owing to demographic changes, and only 4% due to cancer risk changes [32]. In China, it has been postulated that ageing will contribute to a rise in lung, colorectal, and prostate cancer incidences between 2015 and 2030 [33].

### Limitations

First, the current findings depend on quality of incidence data over time. We used the most credible, validated, and IARC-accredited data, sourced locally [34]. Two international indicators of data quality, high morphological verification (MV), and a few death certificate only (DCO) cases in our cancer registry might show variations after 1983 (i.e., MV cases increased from 85% to 96% of MV cases, while DCO cases decreased from 13.3% of cases to 0.4% in the past three decades [30,34]). Relatively higher DCO% could have influenced any apparent upward blip in the disease trend for more deadly cancers without a real change in incidence [35]; however, we did not observe any significant blip in the earlier years during our analysis. Second, due to data availability, this study could not include information on staging at diagnosis, and thus we could not analyse how much of the cancer risk change could be attributable to early or advanced stages. This stopped us from estimating the impact of increasing cancer types on the healthcare system. Third, the current study is descriptive and it is only to speculate about aetiologies of the changes in cancer risk observed. Moreover, we lack cancer subtype information that hinders some analysis. For example, we could not distinguish cardia and non-cardia forms of stomach cancer. Non-cardia stomach cancer is primarily associated with H pylori infection and is likely to account for the changing rates of stomach cancer worldwide [35].

## 5. Conclusions

Demographic changes and ageing in our Chinese population resulted in a marked increase in the number of cancer diagnoses in Hong Kong in the past decades. Continued efforts, such as primary prevention by promoting healthier lifestyles and earlier cancer detection, are needed. Moreover, the surge in incident case counts overall is expected to stress out our healthcare system in terms of the increase in demand of healthcare professionals. Increases in resources and collaboration between disciplinary and healthcare sectors would be necessary for the enhancement of local cancer surveillance and monitoring of cancer disease trends. We also need to rethink how to adjust and enhance our cancer treatment care and cancer surveillance in view of the increased demand from the older patients and the cancer types with fast-increasing incidence rates in our population.

## Data Availability

Publicly available datasets were analysed in this study. The data can be found at Hong Kong Cancer Registry http://www3.ha.org.hk/cancereg/allages.asp (accessed on 1 December 2019), Hong Kong Census and Statistic Department https://www.censtatd.gov.hk/hkstat/sub/sp150.jsp (accessed on 1 December 2019), and Institute for Health Metrics and Evaluation, Global health data exchange http://ghdx.healthdata.org/ (accessed on 1 April 2021).

## Supplementary Materials

The following are available online at https://www.mdpi.com/article/10.3390/cancers13225727/s1, Supplementary Information S1, Supplementary Information S2, Figure S1: Percentage changes in number of cases of the five selected cancers in women and men in Australia, Hong Kong, Japan, Singapore, United Kingdom, United States from 1990 through 2017 attributed to population disease risk and diagnostic practices (i.e., epidemiological component), population size and structure (i.e., demographic component), by sex, using 1990 as the baseline. (N.B. Incidence data are extracted from Global Health Data Exchange).
