# Supplementary material for: Demographic and Epidemiological Contributions to Recent Trends in Cancer Incidence in Hong Kong
Source: Cancers (Basel). 2021 Nov 16;13(22):5727. doi: 10.3390/cancers13225727 (PMC8616530; doi:10.3390/cancers13225727)
Supplement: Supplementary file 1 [file cancers-13-05727-s001.zip › cancers-1428664-supplementary.pdf]

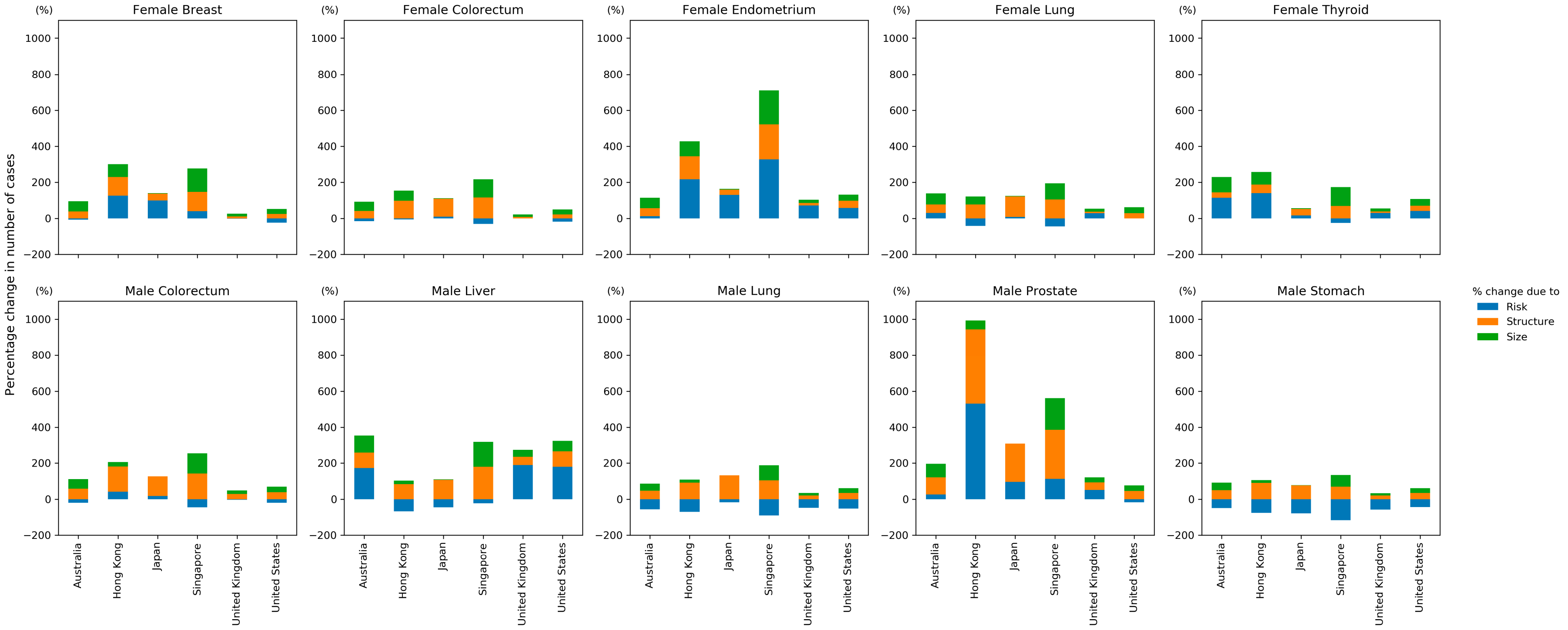

Figure S1. Percentage changes in number of cases of the five selected cancers in women and men in Australia, Hong Kong, Japan, Singapore, United Kingdom, United States from 1990 through 2017 attributed to population disease risk and diagnostic practices (i.e., epidemiological component), population size and structure (i.e., demographic component), by sex, using 1990 as the baseline. (N.B. Incidence data are extracted from Global Health Data Exchange).
