# Supplementary material for: Demographic and Epidemiological Contributions to Recent Trends in Cancer Incidence in Hong Kong
Source: Cancers (Basel). 2021 Nov 16;13(22):5727. doi: 10.3390/cancers13225727 (PMC8616530; doi:10.3390/cancers13225727)
Supplement: Supplementary file 1 [file cancers-13-05727-s001.zip › Supplementary information S1.pdf]

Supplementary information S1

Specifically, in the RiskDiff method, when in the case of making incidence rate decomposition, we consider two time points, t_1 and t_2, with incidence rates s_1 and s_2, say. We decompose the difference of the two incidence rates into disease risk and demographic components. To this end, we calculated the age-standardised incidence rate at time t_1, using population at time t_2 as reference and denote it by s. We can then write

$$s_2 - s_1 = (s_2 - s) + (s - s_1)$$

The first summand can be interpreted as change due to risk factors as they both use the population structure at time t_2 as reference. The second summand can be interpreted as change due to population structure as they both use incidence rates by age group at time t_1. Applying this decomposition to every consecutive year, we apportioned incidence rates into the contribution from the changes in population disease risk and changes in demographic component (i.e., population size and population age structure combined), using year of 1983 (say) as the baseline [13].

For decomposition of percentage change, we divide the above decomposition by s_1.

Similarly, to decompose incident cases, we write

$$c_2 - c_1 = s_2 p_2 - s_1 p_1 = (s_2 - s_1)p_1 + s_2(p_2 - p_1) = (s_2 - s)p_1 + (s - s_1)p_1 + s_2(p_2 - p_1),$$

where c_i is the number of incident cases at time t_i. The first summand can be interpreted as change due to risk factors, the second due to change in population structure, and the third summand due to change in population size. For decomposition of percentage change, we divide the above decomposition by c_1.

Of note, it is possible that the decomposition of relative change of a particular component to show a decrease of more than 100%, but this always comes with, and should be interpreted as offsetting, an increase of more than 100% in some other components. An example is the decomposition of changes in mortality rates in the Global Burden Disease Study 2015 [15].

The online resource developed for this method was listed in the following website in the literature (RiskDiff: rht.iconcologia.net/riskdiff.htm).
