# Supplementary material for: Demographic and Epidemiological Contributions to Recent Trends in Cancer Incidence in Hong Kong
Source: Cancers (Basel). 2021 Nov 16;13(22):5727. doi: 10.3390/cancers13225727 (PMC8616530; doi:10.3390/cancers13225727)
Supplement: Supplementary file 1 [file cancers-13-05727-s001.zip › Supplementary information S2.pdf]

Supplementary information S2

Table. Break down of change in number of cases for the major five cancers in Hong Kong by sex into risk and diagnostic practice, population structure and population size component between year 1983 and 2017, using the Global Burden Disease Study method [15] and the RiskDiff method

	Site Ranked by number of cases in 2017	Component	Change in number of cases (GBD 2015)		Change in number of cases (RiskDiff)	
			Absolute	Relative	Absolute	Relative
**Male**	Overall	**Overall**	8267	96.03%	8267	96.03%
		Risk	-4228	-49.11%	-4198	-48.77%
		Population Structure	10331	120.00%	10275	119.35%
		Population Size	2164	25.13%	2190	25.44%
	Colorectum	**Overall**	2490	306.27%	2490	306.27%
		Risk	472	58.07%	467	57.43%
		Population Structure	1716	211.02%	1707	209.97%
		Population Size	302	37.19%	316	38.87%
	Lung	**Overall**	1193	58.08%	1193	58.08%
		Risk	-1850	-90.08%	-1836	-89.40%
		Population Structure	2541	123.75%	2527	123.05%
		Population Size	501	24.41%	502	24.44%
	Prostate	**Overall**	2077	1274.23%	2077	1274.23%
		Risk	1066	654.32%	1062	651.79%
		Population Structure	907	556.23%	903	553.95%
		Population Size	104	63.69%	112	68.49%
	Liver	**Overall**	340	31.84%	340	31.84%
		Risk	-922	-86.28%	-918	-85.96%
		Population Structure	1014	94.95%	1008	94.42%
		Population Size	247	23.17%	250	23.38%
	Stomach	**Overall**	183	31.61%	183	31.61%
		Risk	-578	99.83%	-576	-99.40%
		Population Structure	638	110.24%	635	109.61%
		Population Size	123	21.20%	124	21.39%
**Female**	Overall	**Overall**	9884	156.52%	9884	156.52%
		Risk	-292	-4.63%	-284	-4.50%
		Population Structure	6070	96.13%	5996	94.95%
		Population Size	4106	65.01%	4172	66.07%
	Breast	**Overall**	3522	413.87%	3522	413.87%
		Risk	1480	173.88%	1461	171.72%
		Population Structure	1224	143.85%	1209	142.10%
		Population Size	818	96.14%	851	100.04%
	Colorectum	**Overall**	1567	204.84%	1567	204.84%
		Risk	-31	-4.01%	-31	-4.01%
		Population Structure	-1000	130.67%	988	129.10%
		Population Size	-598	78.18%	610	79.75%
	Lung	**Overall**	1015	110.81%	1015	110.81%
		Risk	-495	-54.08%	-488	-53.32%
		Population Structure	949	103.57%	937	102.29%
		Population Size	562	61.32%	566	61.84%
	Endometrium	**Overall**	918	581.01%	918	581.01%
		Risk	467	295.47%	461	291.65%
		Population Structure	277	175.42%	274	173.46%
		Population Size	174	110.13%	183	115.90%
	Thyroid	**Overall**	568	420.74%	568	420.74%
		Risk	312	231.09%	309	228.63%
		Population Structure	111	82.11%	109	81.08%
		Population Size	145	107.55%	150	111.03%
